# Skin-Whitening, Antiwrinkle, and Moisturizing Effects of *Astilboides tabularis* (Hemsl.) Engl. Root Extracts in Cell-Based Assays and Three-Dimensional Artificial Skin Models

**DOI:** 10.3390/ijms26125725

**Published:** 2025-06-15

**Authors:** Nam Ho Yoo, Hyun Sook Lee, Sung Min Park, Young Sun Baek, Myong Jo Kim

**Affiliations:** 1 R&D Complex, Kolmar Korea, Seocho-gu, Seoul 06800, Republic of Korea; yoonh@kolmar.co.kr (N.H.Y.);; 2 Department of Bio-Resource Sciences, Kangwon National University, Chuncheon 24341, Republic of Korea; sunnybaek96@naver.com

**Keywords:** *Astilboides tabularis* (Hemsl.) Engl., ethyl acetate, antiwrinkle, moisturizing, skin whitening

## Abstract

This study investigated the potential cosmetic properties of the ethyl acetate (EtOAc) fraction obtained from the roots of *Astilboides tabularis* (Hemsl.) Engl., focusing on skin-whitening, antiwrinkle, and moisturizing effects using cell-based assays and three-dimensional (3D) artificial skin models (Neoderm-ED and Neoderm-ME). The EtOAc fraction showed significant dose-dependent inhibitory activity against tyrosinase (*TYR*) (72.0% inhibition at 50 µg/mL), comparable to that of kojic acid. In α-melanocyte-stimulating hormone (α-MSH)-stimulated Neoderm-ME artificial skin containing melanocytes, the EtOAc fraction reduced melanin synthesis at concentrations of 50 and 75 µg/mL and decreased melanogenesis-related gene expression, including *TYR*, microphthalmia-associated transcription factor (*MITF*), tyrosinase-related protein-1 (*TRP-1*) and *TRP-2*. In the antiwrinkle assays, the EtOAc fraction effectively inhibited elastase activity (41.5% inhibition at 10 µg/mL), exceeding the efficacy of ursolic acid. In the Neoderm-ED artificial skin model, the EtOAc fraction reversed structural damage induced by particulate matter (PM10), restoring epidermal thickness and dermal density. This improvement was supported by the increased expression of skin barrier and antiwrinkle genes, including filaggrin, hyaluronic acid synthase-1 (*HAS-1*), *HAS-2*, aquaporin-3 (*AQP-3*), collagen type I alpha 1 chain (*COL1A1*), elastin, tissue inhibitor of metalloproteinases-1 (*TIMP-1*), and *TIMP-2*, as well as decreased expression of matrix metalloproteinases (*MMP-1*, *MMP-3*, and *MMP-9*). Our results indicate that the EtOAc fraction from *A. tabularis* root has considerable potential as a multifunctional cosmetic.

## 1. Introduction

Recent studies in the fields of cosmetics and dermatology have sought to investigate natural ingredients capable of improving skin health and delaying aging [1]. With the continuous growth of the functional cosmetics market, particularly concerning skin whitening, wrinkle improvement, and moisturizing, studies exploring safe and effective natural substances have become increasingly important [2]. Given the concerns regarding potential skin irritation and adverse effects caused by synthetic whitening and antiaging ingredients currently in use, plant-derived ingredients have received increased attention as viable alternatives [3].

*Astilboides tabularis* (Hemsl.) Engl. is a perennial herbaceous plant belonging to the family Saxifragaceae, primarily distributed in northern temperate regions such as Gangwon Province in Korea and the Jilin and Liaoning provinces of China. It has traditionally been used in Korean folk medicine to treat abdominal pain, enteritis, and diarrhea [4,5], and its young leaves have also been consumed as a seasonal edible vegetable [5]. Until December 2022, it was designated as a Class II endangered wild plant by the Ministry of Environment in Korea due to its limited natural distribution, low regeneration rate, and vulnerability to habitat disturbance caused by illegal harvesting and land development, leading most previous research to focus on its ecological and genetic characteristics, including habitat properties, germination behavior, and environmental adaptability [5,6]. Some studies have also examined propagation methods [7] and reported bioactive compounds. For example, Jin et al. isolated six constituents from the leaves of *A. tabularis* in China [8]. Further pharmacological studies have demonstrated its antioxidant, anti-inflammatory, and anticancer activities [9], and chemical analyses have identified various functional phytochemicals [10]. Although the pharmacological activities of *A. tabularis* have been studied, most investigations have focused on leaf or whole-plant extracts. In contrast, the potential dermatological benefits of its root extract have not yet been systematically examined. This study provides new insights into its skin-related applications, including whitening, antiwrinkle, and moisturizing effects.

Skin whitening is closely associated with the regulation of melanin synthesis, a process governed by various genes, such as tyrosinase (*TYR*), tyrosinase-related protein-1 (*TRP-1*), tyrosinase-related protein-2 (*TRP-2*), and microphthalmia-associated transcription factor (*MITF*) in melanocytes [11]. TYR is a key enzyme that promotes melanin production, and its inhibition represents a crucial mechanism for skin whitening [12]. Additionally, *MITF*, which is activated by exposure to ultraviolet (UV) radiation, increases the expression of *TYR* and *TRP* genes, promoting melanin synthesis; thus, inhibiting this process can reduce skin pigmentation [13].

Wrinkle improvement is closely associated with skin elasticity maintenance through the regulation of collagen and elastin. The key factors promoting wrinkle formation include matrix metalloproteinases (*MMPs*), particularly *MMP-1*, which degrades collagen, and *MMP-3* and *MMP-9*, which facilitate collagen and elastin breakdown [14]. Increased *MMP* activity with aging accelerates collagen and elastin degradation, leading to wrinkle formation. In contrast, tissue inhibitors of metalloproteinases (*TIMPs*), such as *TIMP-1* and *TIMP-2*, inhibit MMP activity and delay wrinkle formation [15]. Therefore, inhibiting *MMP* activity and enhancing *TIMP* expression are essential strategies for wrinkle improvement.

Moisturizing effects are associated with mechanisms that maintain skin hydration and enhance barrier functions. The key elements involved in skin hydration include hyaluronic acid, aquaporin-3 (*AQP-3*), filaggrin, and keratin 1 [16]. The synthesis of hyaluronic acid, which stores water in the dermal and epidermal layers, is promoted by the increased expression of hyaluronan acid synthase 1 (*HAS1*) and hyaluronan acid synthase 2 (*HAS2*) genes [17]. Additionally, *AQP-3* regulates water transport between skin cells, enhancing skin hydration, whereas filaggrin strengthens the skin barrier to prevent moisture loss [18]. Maintaining balance among these factors is crucial for skin hydration.

Therefore, this study was conducted to systematically investigate the effects of the ethyl acetate (EtOAc) fraction derived from a 70% ethanol extract of *A. tabularis* root on skin health. To date, research utilizing the root of this plant for skin-whitening, antiwrinkle, and moisturizing purposes has been extremely limited, and scientific evidence regarding its mechanisms of action remains insufficient. Accordingly, this study aims to evaluate the multifaceted biological activities of this fraction using both cell-based assays and three-dimensional (3D) artificial skin models and to verify its potential as a functional ingredient in cosmetics. Ultimately, this research seeks to expand the scientific utilization of naturally derived ingredients and contribute to the development of eco-friendly and sustainable cosmetic materials. The use of plant-based extracts, rather than synthetic chemicals, reduces environmental impact and aligns with green formulation principles. Furthermore, ethyl acetate, which was used as the main solvent, is recognized for its relatively low toxicity and good biodegradability, and is classified as a green solvent in established solvent selection guides such as CHEM21 and the GSK Solvent Sustainability Index [19].

## 2. Results

### 2.1. TYR Inhibitory Activity

Melanin is synthesized in melanocytes through the enzyme TYR, which catalyzes the conversion of L-tyrosine to L-DOPA and subsequently to L-dopaquinone, a critical rate-limiting step in melanin biosynthesis. TYR inhibition has been widely used to evaluate skin-whitening effects due to melanin pigmentation caused by external factors such as UV radiation [20].

**Figure 1:**
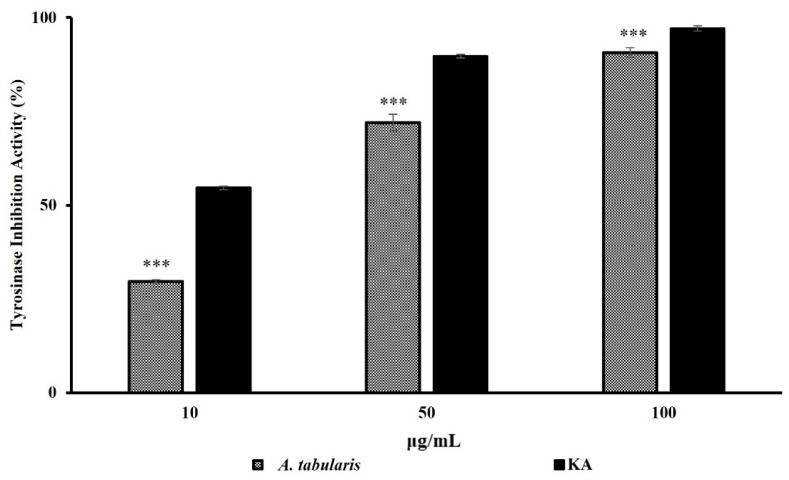
Tyrosinase inhibition assay of the EtOAc fraction from *A. tabularis* root extract. Treatment groups include the EtOAc fraction at concentrations of 10, 50, and 100 µg/mL and kojic acid (KA) as a positive control. Data are presented as the mean ± standard deviation (SD) of three independent experiments (*n* = 3). Statistical significance was determined using a two-tailed unpaired Student’s *t*-test. *** *p* < 0.001, compared with the untreated control.

As shown in Figure 1, the EtOAc fraction of *A. tabularis* root effectively inhibited TYR activity, achieving a high inhibition rate of 72.0% at 50 µg/mL. At 100 µg/mL, its whitening efficacy was comparable to that of kojic acid, a well-known positive control. These findings indicate that the EtOAc fraction effectively inhibits melanin synthesis by suppressing TYR activity, indicating its potential as a whitening agent. Cell viability assays using B16F10 melanoma cells were subsequently performed to determine the appropriate concentrations for further testing.

### 2.2. Elastase Inhibitory Activity

Loss of skin elasticity and wrinkle formation are associated with collagen depletion and elastin degradation. Elastase is an enzyme responsible for elastin degradation, and it hydrolyzes elastin and collagen fibers that contribute to skin elasticity [21]. Therefore, elastase inhibitors play an important role in wrinkle prevention.

**Figure 2:**
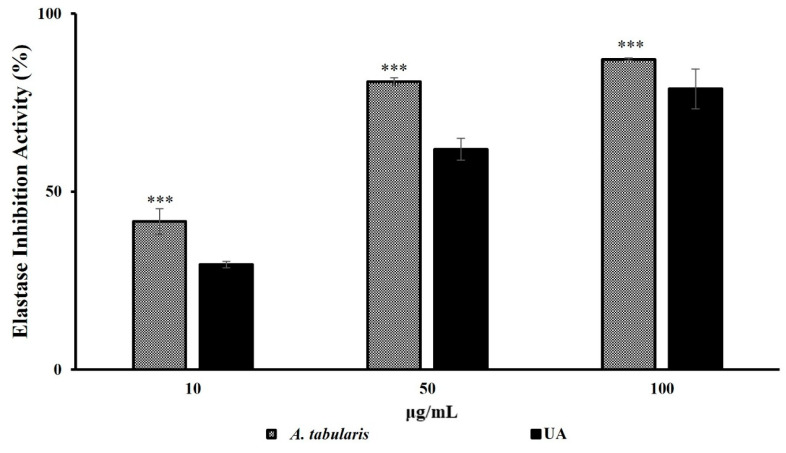
Elastase inhibition assay of the EtOAc fraction from *A. tabularis* root extract. Treatment groups include the EtOAc fraction at concentrations of 10, 50, and 100 µg/mL and ursolic acid (UA) as a positive control. Data are presented as the mean ± standard deviation (SD) of three independent experiments (*n* = 3). Statistical significance was determined using a two-tailed unpaired Student’s *t*-test. *** *p* < 0.001, compared with the untreated control.

*A. tabularis* root EtOAc fraction exhibited potent elastase inhibition, showing 41.5% inhibition even at a low concentration of 10 µg/mL, surpassing the elastase inhibition activity of ursolic acid, a representative elastase inhibitor (Figure 2). The inhibitory activity increased in a dose-dependent manner, highlighting its potential for improving skin elasticity and decreasing wrinkle formation. Subsequent cell viability assays using HaCaT and Detroit 551 cells were performed to establish suitable experimental concentrations.

### 2.3. Cell Viability Assessment

Cell viability experiments using the MTT assay were conducted on B16F10 melanoma cells, HaCaT keratinocytes, and Detroit 551 fibroblast cells to evaluate the cytotoxic effects of the *A. tabularis* root EtOAc fraction and determine optimal experimental concentrations. We found that *A. tabularis* root EtOAc fraction increased cytotoxicity in a dose-dependent manner, with treatment concentrations ranging from 10 to 200 µg/mL.

**Figure 3:**
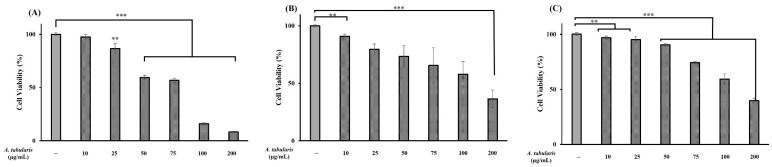
Inhibitory effect of the EtOAc fraction from *A. tabularis* root extract on cytotoxicity in three cell lines: (**A**) B16F10 (murine metastatic melanoma), (**B**) HaCaT (human immortalized keratinocyte), and (**C**) Detroit 551 (human skin fibroblasts). The treatment groups include the EtOAc fraction at concentrations of 10, 25, 50, 75, 100, and 200 µg/mL. Data are presented as the mean ± standard deviation (SD) of three independent experiments (*n* = 3). Statistical significance was determined using a two-tailed unpaired Student’s *t*-test. ** *p* < 0.01, *** *p* < 0.001, compared with the untreated control.

B16F10 cells exhibited the highest sensitivity (Figure 3A), with viability decreasing sharply from 56.7% at 75 µg/mL to 15.9% at 100 µg/mL. Thus, 75 µg/mL was selected as the maximum concentration for subsequent experiments. HaCaT cells exhibited a viability of 57.9% at 100 µg/mL, dropping significantly to 36.5% at 200 µg/mL. Hence, 100 µg/mL was set as the highest experimental concentration (Figure 3B). Similarly, the Detroit 551 cells showed viability rates of 59.3% and 39.9% at 100 and 200 µg/mL, respectively, for which 100 µg/mL was also established as the maximum experimental concentration (Figure 3C). Collectively, experimental concentrations up to 75 µg/mL were determined suitable for further analyses.

### 2.4. Melanin Production Inhibition in B16F10 Cells

**Figure 4:**
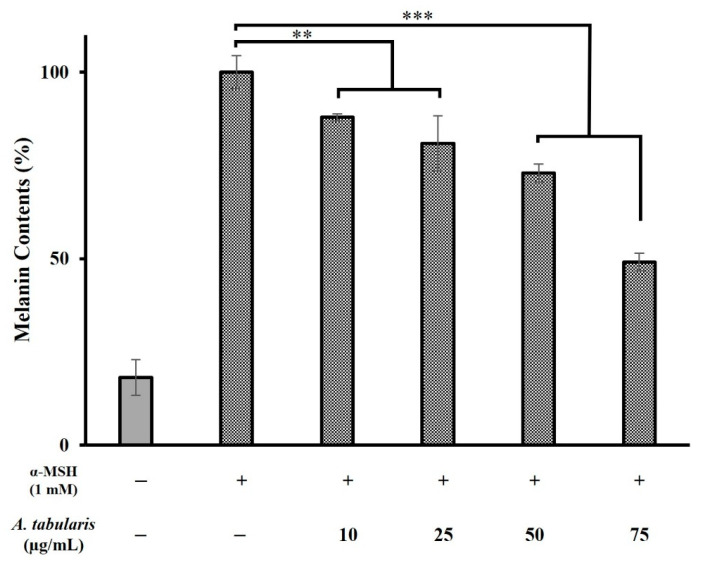
Effect of the EtOAc fraction from *A. tabularis* root extract on melanin content in B16F10 cells. Treatment groups include the EtOAc fraction at concentrations of 10, 25, 50, and 75 µg/mL. Data are presented as the mean ± standard deviation (SD) of three independent experiments (*n* = 3). Statistical significance was determined using a two-tailed unpaired Student’s *t*-test. ** *p* < 0.01, *** *p* < 0.001, compared with the untreated control.

Melanin-producing cells are activated upon exposure to stimuli, such as UV radiation. Melanin biosynthesis involves various enzymes, primarily TYR, and is regulated by these enzymes under external stimulation [22,23,24]. To evaluate whitening efficacy, α-MSH, a melanin-stimulating hormone, was used to induce melanin production. Treatment with α-MSH alone significantly increased the melanin content, whereas treatment with *A. tabularis* root EtOAc fraction inhibited melanin production in a dose-dependent manner. Specifically, the fraction exhibited inhibition rates of 27.1% and 51.0% at 50 and 75 µg/mL, respectively (Figure 4).

### 2.5. mRNA Expression Levels of Whitening-Related Factors in B16F10 Melanoma Cells

During melanogenesis, *MITF* is crucial for regulating the expression of *TYR*, *TRP-1*, and *TRP-2* [13]. *TYR* converts L-tyrosine to L-DOPA and subsequently to dopaquinone, which are essential steps in melanin synthesis [12]. *TRP-1* and *TRP-2* convert 5,6-dihydroxyindole-2-carboxylic acid into indole-5,6-quinone-2-carboxylic acid, ultimately synthesizing melanin [25].

**Figure 5:**
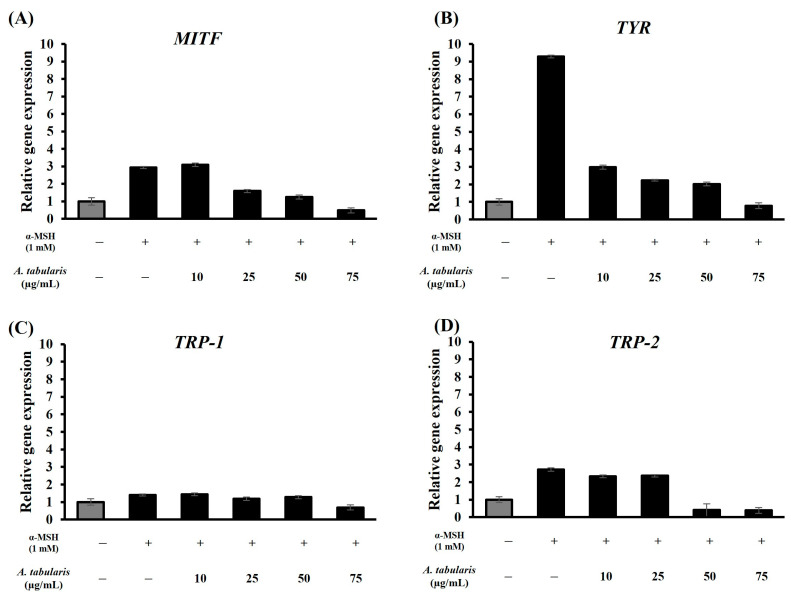
mRNA expression of EtOAc fraction from *A. tabularis* root extract in α-MSH-stimulated B16F10 cells. (**A**) *MITF*, (**B**) *TYR*, (**C**) *TRP-1*, and (**D**) *TRP-2*. Each data point is presented as the mean ± standard deviation of three replicate experiments.

To examine the effects of *A. tabularis* root EtOAc fraction on melanin-related gene expression, RT-PCR was performed on *TYR*, *MITF*, *TRP-1*, and *TRP-2* following α-MSH stimulation (Figure 5A–D). Compared with the α-MSH-untreated group, cells treated with α-MSH exhibited significantly elevated expression of melanin synthesis genes, which was effectively suppressed by the EtOAc fraction in a dose-responsive manner. *TYR* showed the most pronounced inhibition (Figure 5B), and at 75 µg/mL, gene expression levels were comparable to those of the α-MSH-untreated control.

**Figure 6:**
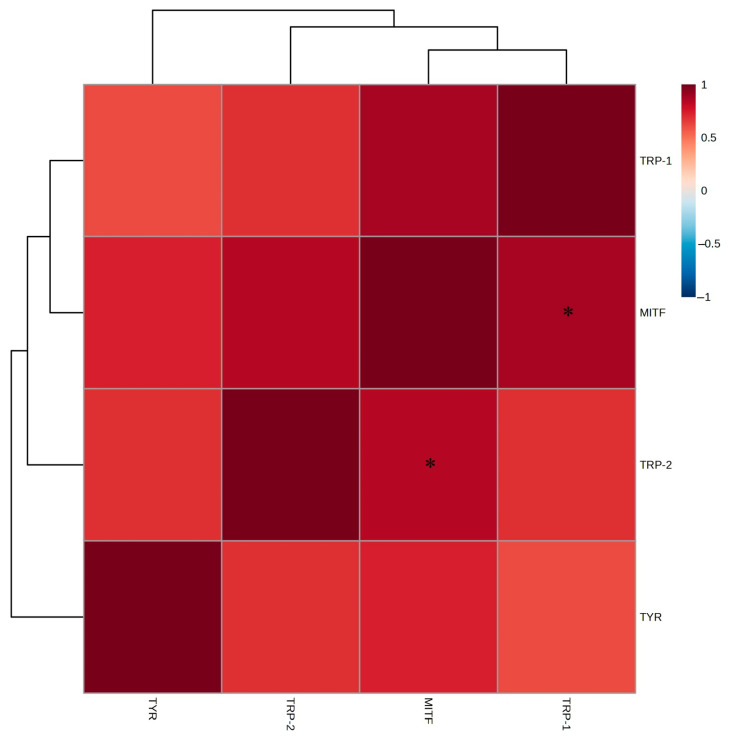
Correlation analysis in mRNA expression of the EtOAc fraction from *A. tabularis* root extract in α-MSH-stimulated B16F10 cells. The correlation between the respective genes was analyzed. Superscripts mean significant differences between the experimental groups, as determined by an independent sample *t*-test (* *p* < 0.05).

Strong positive correlations were observed between the EtOAc fraction and *TYR*, *MITF*, *TRP-1*, and *TRP-2*, with *TYR* showing the highest inhibition rate, *MITF* and *TRP-1* demonstrating similar inhibitory patterns, and *TRP-2* exhibiting relatively higher inhibition than *TRP-1* (Figure 6). These findings indicate that the EtOAc fraction affects multiple genes involved in melanin biosynthesis. Thus, the whitening effects of *A. tabularis* root EtOAc fraction involve broad suppression of the melanogenesis pathway, highlighting *TYR* downregulation as a critical mechanism.

### 2.6. mRNA Expression Levels of Moisturizing Factors in HaCaT Cells

**Figure 7:**
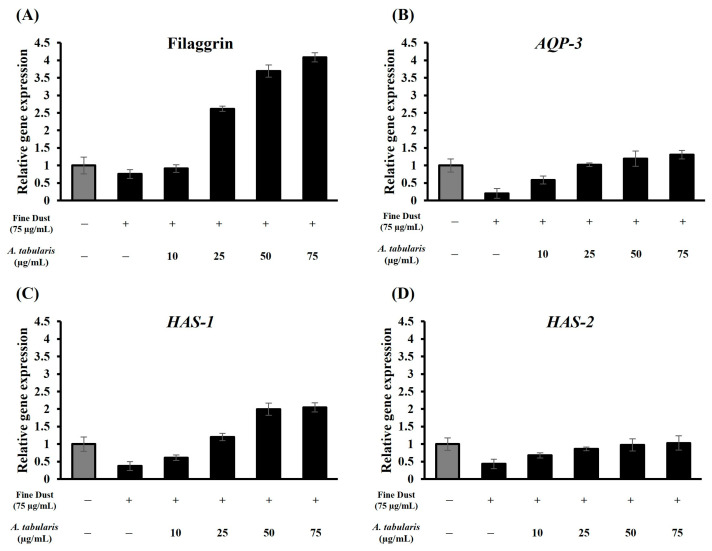
mRNA expression of EtOAc fraction from *A. tabularis* root extract in fine dust-stimulated HaCaT cells. (**A**) Filaggrin, (**B**) *AQP-3*, (**C**) *HAS-1*, and (**D**) *HAS-2*. Each data point is presented as the mean ± standard deviation of three replicate experiments.

To evaluate the skin moisturizing efficacy of *A. tabularis* root EtOAc fraction, expression levels of key moisturizing-related genes, including filaggrin, *AQP-3*, *HAS-1*, and *HAS-2*, were analyzed via RT-PCR in HaCaT cells (Figure 7A–D). Under skin barrier damage conditions induced by fine dust (75 µg/mL), treatment with the *A. tabularis* root EtOAc fraction led to a dose-dependent upregulation of all analyzed genes.

**Figure 8:**
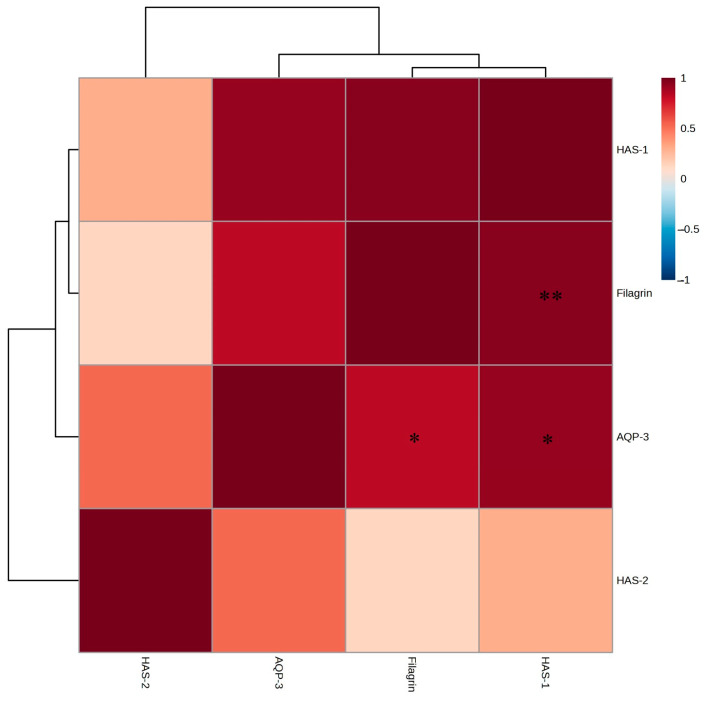
Correlation analysis in mRNA expression of EtOAc fraction from *A. tabularis* root extract in fine dust-stimulated HaCaT cells. The correlation between the respective genes was analyzed. Superscripts mean significant differences between the experimental groups, as determined by an independent sample *t*-test (* *p* < 0.05, ** *p* < 0.01).

Notably, filaggrin and *HAS-1* exhibited the most significant increases in expression levels, with *AQP-3* and *HAS-2* also promoting significant increases (Figure 7A–D). Strong positive correlations were observed between the *A. tabularis* root EtOAc fraction and filaggrin, *AQP-3*, *HAS-1*, and *HAS-2* (Figure 8). These results indicate that the EtOAc fraction of *A. tabularis* root effectively influences the expression of key genes involved in skin hydration and barrier formation.

These findings indicate that the EtOAc fraction promotes hydration and actively enhances the skin barrier and hydration by increasing the expression of crucial moisturizing factors. Filaggrin increases water retention capacity within the stratum corneum [26], whereas *AQP-3* regulates water and glycerol transport, which are crucial for maintaining skin hydration and elasticity [27]. *HAS-1* and *HAS-2* control hyaluronic acid synthesis, which is essential for skin hydration [28]. The present study highlights the potential of the *A. tabularis* root EtOAc fraction as a promising natural moisturizer and skin protectant.

### 2.7. mRNA Expression Levels of Wrinkle-Related Factors in Detroit 551 Cells

**Figure 9:**
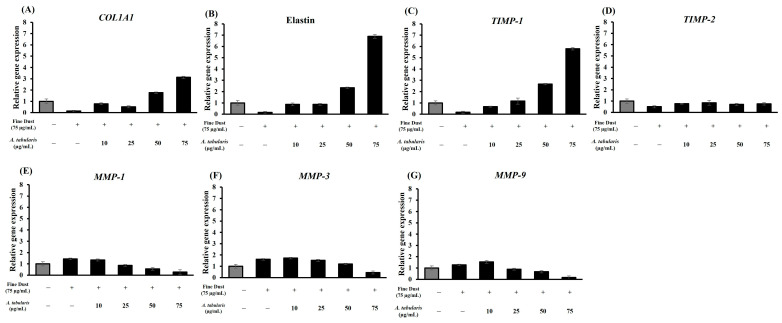
mRNA expression of EtOAc fraction from *A. tabularis* root extract in fine dust-stimulated Detroit 551 cells. (**A**) *COL1A1*, (**B**) elastin, (**C**) *TIMP-1*, (**D**) *TIMP-2*, (**E**) *MMP-1*, (**F**) *MMP-3*, and (**G**) *MMP-9*. Each data is presented as the means ± standard deviation of three replicate experiment.

To evaluate the effects on wrinkle improvement, mRNA expression of the wrinkle-related genes, including *COL1A1*, elastin, *TIMP-1*, *TIMP-2*, *MMP-1*, *MMP-3*, and *MMP-9*, was analyzed by RT-PCR using Detroit 551 cells (Figure 9A–G). Skin aging and wrinkle formation were induced using fine dust (75 µg/mL). The expression of the wrinkle-preventing genes *COL1A1*, elastin, *TIMP-1*, and *TIMP-2* increased in a dose-dependent manner, whereas the expression of the wrinkle-promoting genes *MMP-1*, *MMP-3*, and *MMP-9* decreased at higher treatment concentrations.

**Figure 10:**
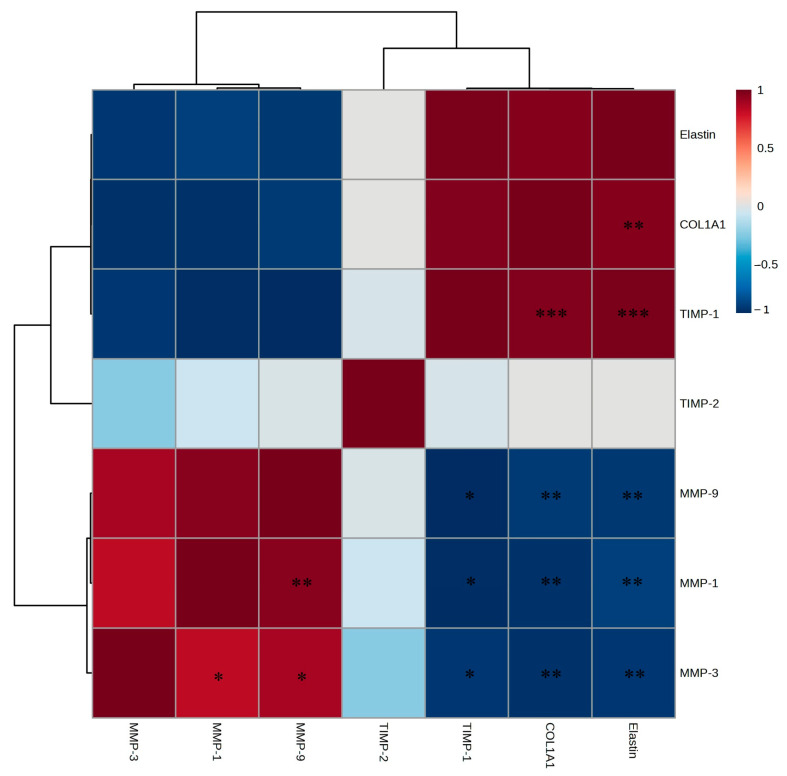
Correlation analysis of mRNA expression of the EtOAc fraction from *A. tabularis* root extract in fine dust-stimulated Detroit 551 cells. The correlation was analyzed between the respective genes. Superscripts mean significant difference between the experimental groups, as determined by an independent sample *t*-test (* *p* < 0.05, ** *p* < 0.01, *** *p* < 0.001).

Notably, the expression of *COL1A1* and elastin exhibited the most significant increases, with *TIMP-1* also showing a significant elevation. Conversely, the expression of *MMP*-related genes was inhibited with increasing EtOAc fraction concentrations. The EtOAc fraction exhibited strong positive correlations with *COL1A1*, elastin, *TIMP-1*, and *TIMP-2* and negative correlations with *MMP-1*, *MMP-3*, and *MMP-9* (Figure 10). These findings indicate that the EtOAc fraction of *A. tabularis* root effectively regulates the genes associated with skin aging and wrinkle formation.

These results indicate that the mechanisms by which the *A. tabularis* fraction ameliorates skin aging and wrinkle formation extend beyond simply inhibiting *MMP* to promoting collagen (*COL1A1*) and elastin synthesis and regulating *MMP*-mediated degradation via *TIMP* activation. Collagen provides structural support [29], whereas elastin maintains skin elasticity [30]. Furthermore, *TIMP-1* and *TIMP-2* inhibited *MMP* activity, reducing collagen and elastin degradation and delaying wrinkle formation [31]. Thus, the *A. tabularis* root EtOAc fraction exhibits potential as a natural antiaging ingredient.

### 2.8. Skin Irritation Test Using Artificial Skin Models

**Figure 11:**
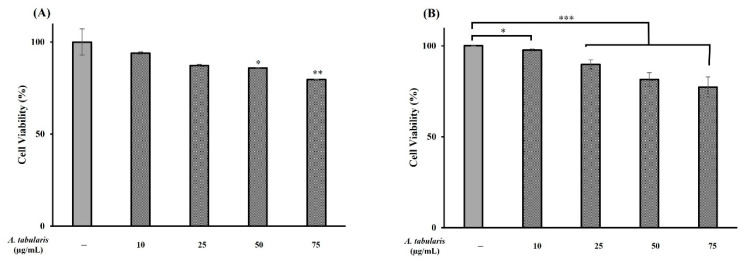
Inhibitory effect of the EtOAc fraction from *A. tabularis* root extract on cytotoxicity in artificial skin tissues: Neoderm-ED ((**A**): epidermis + dermis) and Neoderm-ME ((**B**): epidermis + melanocytes). Treatment groups include the EtOAc fraction at concentrations of 10, 25, 50, and 75 µg/mL. Data are presented as the mean ± standard deviation (SD) of three independent experiments (*n* = 3). Statistical significance was determined using a two-tailed unpaired Student’s *t*-test. * *p* < 0.05, ** *p* < 0.01, *** *p* < 0.001, compared with the untreated control.

Based on the results of our in vitro experiments, which demonstrated the potential whitening and antiwrinkle effects of the *A. tabularis* root EtOAc fraction, primary irritation was further assessed using artificial skin models Neoderm-ED (epidermis + dermis) and Neoderm-ME (epidermis + melanocytes). Treatment with 75 µg/mL of the *A. tabularis* root EtOAc fraction induced a cell viability of 79.7% for Neoderm-ED (Figure 11A) and 77.4% for Neoderm-ME (Figure 11B), indicating low irritation potential and confirming its suitability for skin applications.

### 2.9. Fine Dust-Stimulated Skin Damage and Recovery Analysis in the Neoderm-ED Artificial Skin Model

**Figure 12:**
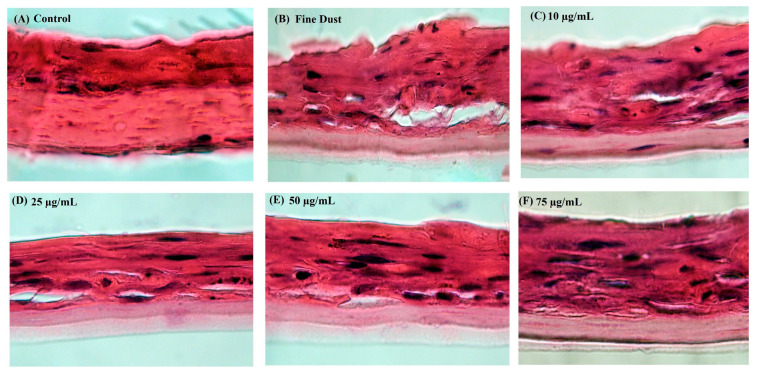
Histological analysis of the EtOAc fraction from *A. tabularis* root extract in fine dust-stimulated 3D artificial skin tissue (Neoderm-ED). Hematoxylin and eosin (H&E) staining images were acquired at 1000× magnification. (**A**) Untreated control (negative control), (**B**) fine dust-stimulated only (positive control), (**C**) fine dust + EtOAc 10 µg/mL, (**D**) fine dust + EtOAc 25 µg/mL, (**E**) fine dust + EtOAc 50 µg/mL, (**F**) fine dust + EtOAc 75 µg/mL. Data represent one of three independent experiments (*n* = 3). Morphological improvement in tissue structure was evaluated qualitatively.

The current study evaluated the restorative effects of the *A. tabularis* root EtOAc fraction on fine dust-induced skin damage using the Neoderm-ED artificial skin model, which consists of epidermal and dermal layers (Figure 12A–F). Histological analysis using H&E staining was conducted to observe structural changes in the untreated control, the group treated with fine dust, and the group treated with fine dust plus various concentrations (10, 25, 50, and 75 µg/mL) of the EtOAc fraction.

As shown in Figure 12A, the untreated skin equivalents, which served as the negative control group, maintained normal tissue architecture. In contrast, the fine dust-treated group (Figure 12B) exhibited epidermal damage, irregular stratum corneum formation, and reduced dermal density. However, treatment with the EtOAc fraction (Figure 12C–F) led to gradual restoration of skin structure, with marked improvements in epidermal thickness and dermal density observed at 50 and 75 µg/mL. These results indicate that the EtOAc fraction effectively mitigates fine dust-induced skin damage and enhances skin barrier recovery. Consequently, the *A. tabularis* root EtOAc fraction shows potential as a restorative functional cosmetic ingredient that protects against environmental pollutants.

### 2.10. Analysis of the Skin Protection and Antiwrinkle Mechanisms in the Neoderm-ED Model

**Figure 13:**
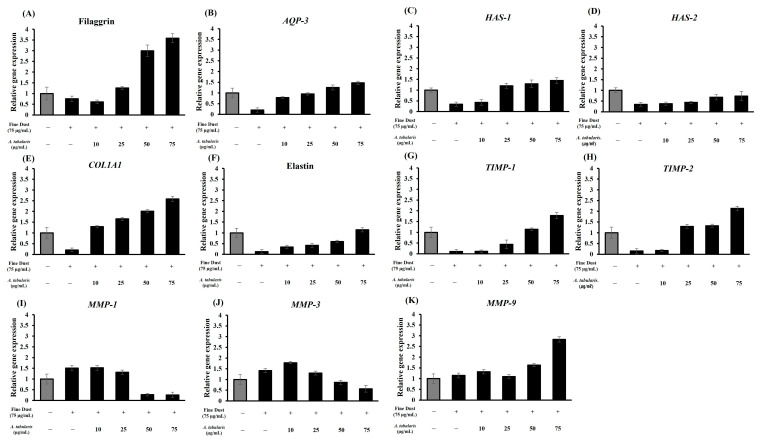
mRNA expression of the EtOAc fraction from *A. tabularis* root extract in fine dust-stimulated artificial skin tissue Neoderm-ED. (**A**) Filaggrin, (**B**) *AQP-3*, (**C**) *HAS-1*, (**D**) *HAS-2*, (**E**) *COL1A1*, (**F**) elastin, (**G**) *TIMP-1*, (**H**) *TIMP-2*, (**I**) *MMP-1*, (**J**) *MMP-3*, and (**K**) *MMP-9*. Each data point is presented as the mean ± standard deviation of three replicate experiments.

To confirm the mechanisms underlying the observed protective, moisturizing, and antiwrinkle effects identified through histological analysis, RT-PCR was performed to measure the expression of genes related to skin hydration, barrier function, and wrinkle formation, including filaggrin, *AQP-3*, *HAS-1*, *HAS-2*, *COL1A1*, *elastin*, *TIMP-1*, *TIMP-2*, *MMP-1*, *MMP-3*, and *MMP-9* (Figure 13A–K). Accordingly, we found a dose-dependent increase in the expression of genes involved in moisturizing and skin barrier enhancement (filaggrin, *AQP-3*, *HAS-1*, *HAS-2*) and wrinkle prevention (*COL1A1*, elastin, *TIMP-1*, *TIMP-2*) but a decrease in the expression of *MMP-1*, *MMP-3*, and *MMP-9*. Notably, filaggrin, *HAS-1*, *AQP-3*, *TIMP-1*, and *COL1A1* showed the most significant increases, whereas *MMP*-related genes were notably suppressed at higher concentrations.

**Figure 14:**
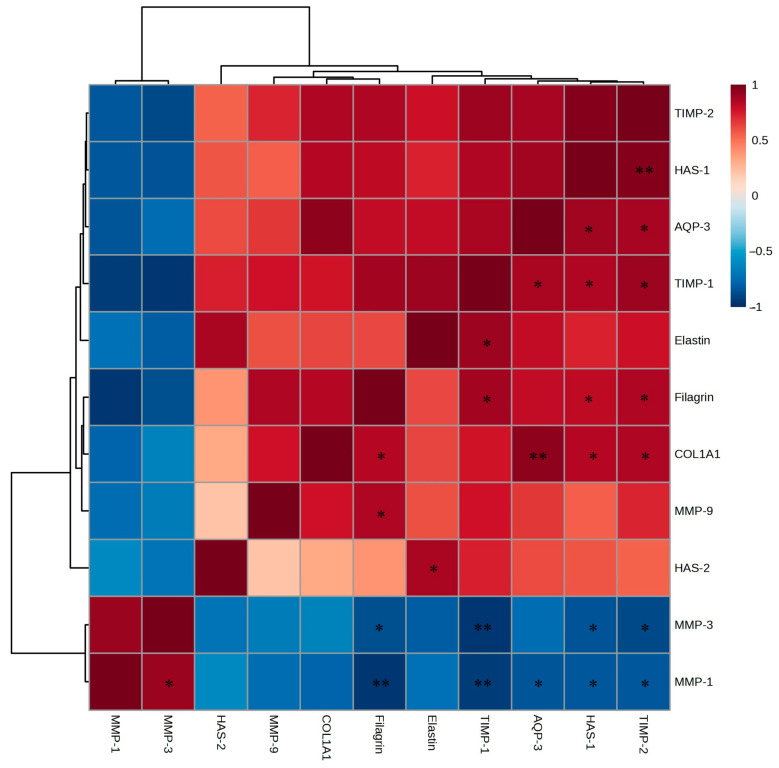
Correlation analysis in mRNA expression of the EtOAc fraction from *A. tabularis* root extract in fine dust-stimulated artificial skin tissue Neoderm-ED. The correlation between the respective genes was analyzed. Superscripts mean significant differences between the experimental groups, as determined by an independent sample *t*-test (* *p* < 0.05, ** *p* < 0.01).

Correlation analysis (Figure 14) indicated that the EtOAc fraction showed strong positive correlations with filaggrin, *AQP-3*, *HAS-1*, *HAS-2*, *COL1A1*, and elastin, which are essential for maintaining skin moisture and elasticity, and negative correlations with *MMP-1*, *MMP-3*, and *MMP-9*. These results suggest that the EtOAc fraction promotes skin hydration and barrier formation while inhibiting *MMP* activity, thereby protecting the skin and decreasing wrinkles.

The mechanisms involve filaggrin-induced enhancement of natural moisturizing factor formation within the stratum corneum to strengthen the skin barrier [32] and *AQP-3*-facilitated water and glycerol transport to improve skin hydration and elasticity. Additionally, *HAS-1* and *HAS-2* increase hyaluronic acid synthesis, which is essential for skin hydration, whereas *COL1A1* and elastin maintain structural integrity and elasticity. The reduction in *MMP-1*, *MMP-3*, and *MMP-9* expression is consistent with enhanced skin protection, while the increase in *TIMP-1* and *TIMP-2* inhibits collagen and elastin degradation by suppressing *MMP* activity. These findings demonstrate that the *A. tabularis* root EtOAc fraction effectively promotes moisturizing, barrier function, and antiwrinkle effects in artificial skin models, underscoring its potential as a natural protective and antiaging cosmetic ingredient.

### 2.11. Analysis of Melanin Inhibitory Effects in the Neoderm-ME Artificial Skin Model

**Figure 15:**
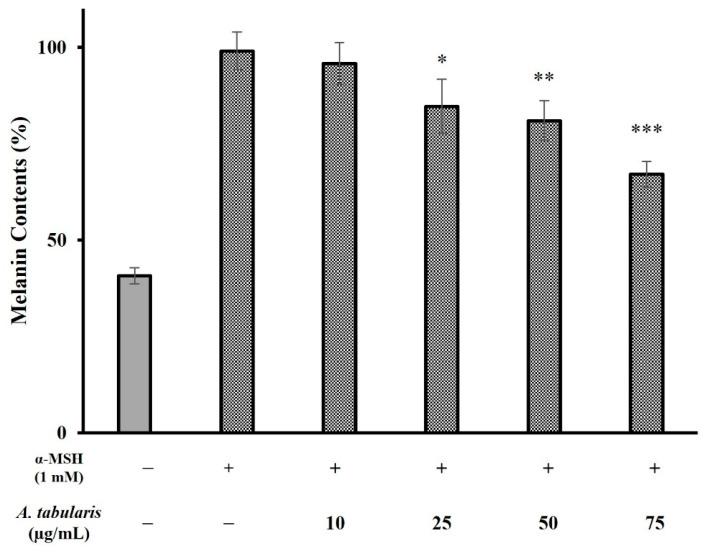
Effect of the EtOAc fraction from *A. tabularis* root extract on melanin content in the artificial skin tissue model Neoderm-ME. Treatment groups include the EtOAc fraction at concentrations of 10, 25, 50, and 75 µg/mL. Data are presented as the mean ± standard deviation (SD) of three independent experiments (*n* = 3). Statistical significance was determined using two-tailed unpaired Student’s *t*-test. * *p* < 0.05, ** *p* < 0.01, *** *p* < 0.001, compared with the untreated control.

To assess the melanin inhibitory effects of the *A. tabularis* root EtOAc fraction, we used the Neoderm-ME artificial skin model, which consists of the epidermis and melanocytes. Melanin synthesis was induced using α-MSH (1 mM), after which the artificial skin was treated with various concentrations of the EtOAc fraction (10, 25, 50, and 75 µg/mL) and then analyzed for melanin content (Figure 15).

Our results showed a significant increase in melanin production in the α-MSH-treated control group compared to untreated controls. Treatment with the EtOAc fraction dose dependently reduced melanin production. Specifically, melanin synthesis was significantly reduced at concentrations of 50 and 75 µg/mL, with the highest inhibition observed at 75 µg/mL (Figure 15). These results indicate that the *A. tabularis* root EtOAc fraction effectively modulates melanin synthesis, suggesting its potential as a natural skin-whitening ingredient.

### 2.12. Analysis of Whitening-Related Gene Expression and Mechanisms in the Neoderm-ME Model

**Figure 16:**
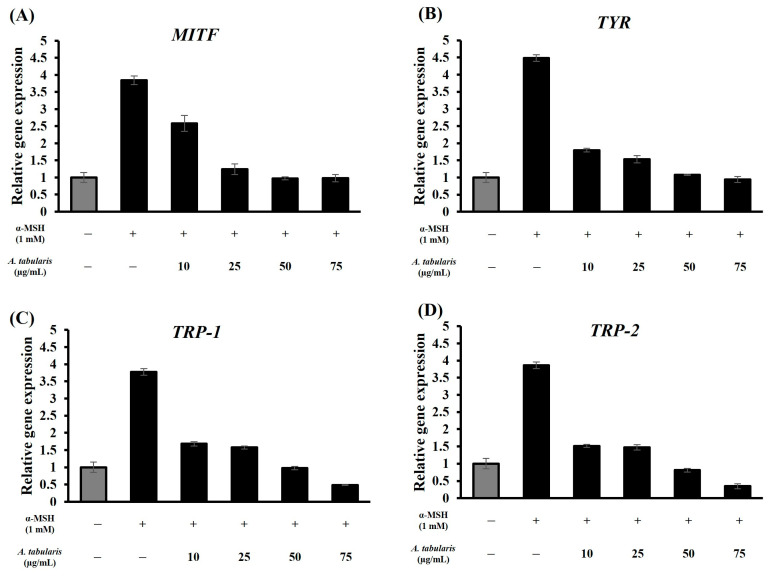
mRNA expression of the EtOAc fraction from *A. tabularis* root extract in α-MSH-stimulated artificial skin tissue Neoderm-ME. (**A**) *MITF*, (**B**) *TYR*, (**C**) *TRP-1*, and (**D**) *TRP-2*. Each data point is presented as the mean ± standard deviation of three replicate experiments.

To investigate the molecular mechanisms underlying the whitening effects of the *A. tabularis* root EtOAc fraction, we analyzed the expression of melanogenesis-related genes *MITF*, *TYR*, *TRP-1*, and *TRP-2* using RT-PCR (Figure 16A–D). All evaluated genes showed a dose-dependent reduction in expression. Notably, *TYR* expression exhibited the strongest inhibition, followed by comparable inhibition rates for *MITF* and *TRP-1*, with *TRP-2* displaying relatively higher inhibition.

**Figure 17:**
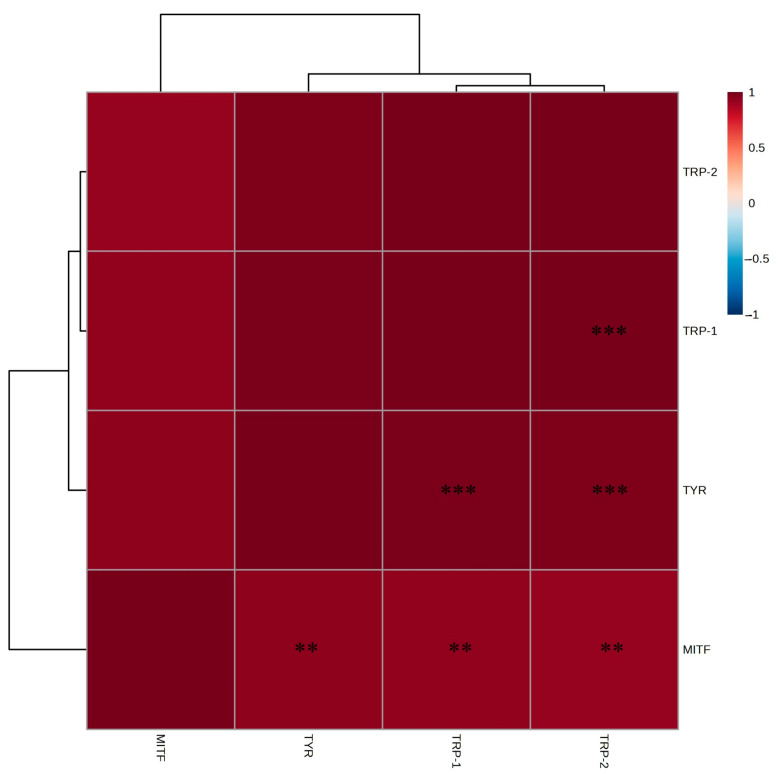
Correlation analysis in mRNA expression of the EtOAc fraction from *A. tabularis* root extract in fine dust-stimulated artificial skin tissue Neoderm-ME. The correlation between the respective genes was analyzed. Superscripts mean significant difference between the experimental groups, as indicated by an independent sample *t*-test ** *p* < 0.01, *** *p* < 0.001).

Correlation analysis (Figure 17) revealed strong positive correlations between the EtOAc fraction and *MITF*, *TYR*, *TRP-1*, and *TRP-2*. These results demonstrate that the EtOAc fraction influences multiple melanogenesis-related genes, suggesting a comprehensive inhibitory effect throughout the melanin biosynthetic pathway. Therefore, the current study suggests that the *A. tabularis* root EtOAc fraction could be a promising, safe, and effective natural whitening agent.

## 3. Discussion

This study evaluated the effects of the *A. tabularis* (Hemsl.) Engl. root EtOAc fraction on skin whitening, wrinkle improvement, and moisturization. The experiments were conducted using both traditional cell culture assays and advanced 3D artificial skin models (Neoderm-ED and Neoderm-ME) to better demonstrate the potential for practical skin applications.

Initially, the whitening efficacy of *A. tabularis* root EtOAc fraction was assessed using TYR inhibitory activity and melanin synthesis inhibition assays. We found that the EtOAc fraction remarkably inhibited TYR by 72.0% at a concentration of 50 µg/mL, which is comparable to that of the conventional whitening agent kojic acid. Additionally, experiments using B16F10 melanoma cells demonstrated a dose-dependent reduction in melanin production, accompanied by a decrease in the expression of melanogenesis-related genes, including *MITF*, *TYR*, *TRP-1*, and *TRP-2*.

To support these cell-based findings, the Neoderm-ME artificial skin model (epidermis and melanocytes) was used. Treatment with *A. tabularis* root EtOAc fractions (10, 25, 50, and 75 µg/mL) significantly reduced α-MSH-induced melanin synthesis in a concentration-dependent manner, with the most pronounced inhibition observed at 75 µg/mL. RT-PCR analysis further confirmed the suppression of melanogenesis-related gene expression, highlighting the strong inhibitory effect of *TYR* expression. Given that *MITF* regulates key melanin-related genes (*TYR*, *TRP-1*, and *TRP-2*), these results indicate that the EtOAc fraction exerts comprehensive control over the melanogenesis pathway, supporting its potential as a natural whitening ingredient. However, as our mechanistic validation is currently limited to mRNA expression, further studies involving protein-level analyses such as Western blotting or ELISA are necessary to confirm these regulatory effects at the translational level.

Elastase inhibitory assays conducted to determine the wrinkle-improving effects of the EtOAc fraction revealed significant elastase inhibition (41.5%), even at a low concentration of 10 µg/mL, surpassing that of ursolic acid, a known elastase inhibitor. Gene expression analyses also indicated an increase in the expression of the wrinkle-preventive genes, including *COL1A1*, elastin, *TIMP-1,* and *TIMP-2*, and a decrease in the expression of *MMP-1*, *MMP-3*, and *MMP-9*, involved in matrix degradation.

To confirm these protective and antiwrinkle effects, the Neoderm-ED artificial skin model (epidermis and dermis layers) was used, with skin damage being induced through fine dust exposure. Although the fine dust-treated control showed epidermal damage, irregular stratum corneum formation, and reduced dermal density, the treatment groups receiving the *A. tabularis* root EtOAc fraction showed a clear recovery of epidermal thickness and dermal tissue integrity, particularly at higher concentrations (50 and 75 µg/mL). Moreover, significant increases in the expression of moisturizing-related genes (filaggrin, *HAS-1*, *HAS-2*, and *AQP-3*) and structural proteins (*COL1A1* and elastin) were observed, accompanied by a decrease in *MMP-1*, *MMP-3*, and *MMP-9* expression. These findings strongly indicate that the EtOAc fraction promotes moisture, enhances the skin barrier, and has significant antiaging properties.

Additionally, artificial skin irritation tests performed using the Neoderm-ED and Neoderm-ME models showed that treatment with the EtOAc fraction at 75 µg/mL resulted in cell viabilities of 79.7% and 77.4%, respectively, confirming its low skin irritation potential and suitability for topical application.

Our phytochemical profiling using LC-MS/MS and HPLC (Supplementary Figures S1 and S2 and Table S1) confirmed bergenin and gallic acid as major constituents in the EtOAc fraction. Previous research identified bergenin and gallic acid as the main marker compounds in the *A. tabularis* root EtOAc fraction [33]. Bergenin, a C-glycoside of 4-O-methyl gallic acid, is commonly found in Bergenia species [34], while gallic acid (3,4,5-trihydroxybenzoic acid) is a widespread phenolic phytochemical [35]. Both compounds have been reported to co-exist in plants such as Bergenia ligulata [36].

However, our bioactivity results suggest that the efficacy of the EtOAc fraction cannot be solely attributed to these two compounds. While both gallic acid and bergenin are reported to possess individual whitening and antiaging activities, the enhanced biological responses—such as tyrosinase inhibition, MMP suppression, collagen promotion, and moisturizing gene upregulation—were observed only when the full EtOAc fraction was applied. These effects likely stem from the complex synergistic interactions among multiple constituents, including unidentified minor compounds. Thus, our findings reinforce the notion that the complete phytochemical matrix of *A. tabularis* root EtOAc extract contributes to its multifunctional skin benefits.

Gallic acid is well documented for its skin-whitening and antiaging effects, operating via mechanisms including potent tyrosinase inhibition, regulation of melanogenesis pathways, antioxidant activity, MMP/elastase inhibition, and collagen synthesis stimulation [37,38,39,40,41]. Furthermore, bergenin derivatives, particularly when esterified with phenolic acids such as gallic acid, show potential, suggesting a possible synergistic effect when these two compounds are present together [42]. However, the novelty of this study extends beyond the individual contributions of these well-characterized components. While gallic acid and bergenin are significant, the observed multi-faceted beneficial effects of the *A. tabularis* EtOAc fraction—encompassing comprehensive skin whitening, significant wrinkle improvement, and enhanced moisturization across both cellular and advanced 3D skin models—suggest a synergistic action of its complex phytochemical profile. This holistic activity potentially offers a more robust and complete solution for skin care compared to isolated compounds.

Many cosmetic agents rich in phenolics primarily target one or two pathways. For instance, arbutin or kojic acid are known for their singular tyrosinase inhibitory effects, while certain peptides focus on collagen synthesis. In contrast, our findings demonstrate that the *A. tabularis* EtOAc fraction simultaneously influences multiple interconnected pathways: inhibiting tyrosinase, regulating melanin-related gene expression, suppressing elastase and MMPs, and upregulating genes crucial for collagen, elastin, and moisturization (e.g., filaggrin, *HAS-1*, *HAS-2*, and *AQP-3*). This broad spectrum of activity, particularly as validated in sophisticated 3D artificial skin models that closely mimic human skin physiology, represents a key differentiation and provides novel insights into the practical efficacy of this specific plant extract.

Compared to *Centella asiatica*, a widely utilized botanical known for its wound healing, antioxidant, and antiwrinkle properties mediated by triterpenoids such as asiaticoside and madecassoside [43,44], the *A. tabularis* EtOAc fraction demonstrated comparable or superior effects on melanogenesis inhibition, skin barrier restoration, and collagen-related gene regulation, particularly in advanced 3D skin models. This suggests its potential as a multifunctional and eco-friendly alternative to conventional plant-based actives in dermocosmetic formulations.

Furthermore, while other phenolic-rich extracts exist, the unique combination and concentration of active compounds within the *A. tabularis* EtOAc fraction may contribute to its superior efficacy and lower irritation potential observed in this study. For example, while some plant extracts may exhibit anti-melanogenic properties, they may lack the comprehensive antiwrinkle or moisturizing effects demonstrated here, or vice-versa. Future studies involving chemical profiling and bioactivity-guided fractionation could further elucidate the precise molecular interplay among the compounds responsible for the observed synergistic effects, providing a deeper understanding of its unique cosmetic potential.

**Figure 18:**
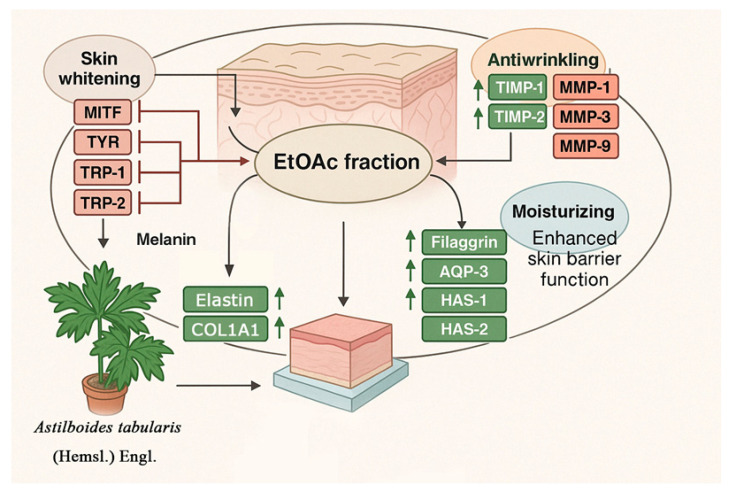
Proposed mechanisms of skin-whitening, antiwrinkle, and moisturizing effects of the EtOAc fraction from *A. tabularis* root extract.

Collectively, these findings suggest that the EtOAc fraction modulates multiple skin-related pathways to exert skin-whitening, antiwrinkle, and moisturizing effects. The proposed mechanisms underlying these effects are illustrated in Figure 18.

## 4. Materials and Methods

### 4.1. Astilboides tabularis Root Extract and Fractions

The roots of *Astilboides tabularis* (Hemsl.) Engl. used in this study were sourced from the Gangwon Nature Environment Research Park (Hongcheon, Republic of Korea; coordinates: 37.947479° N, 127.754712° E). All plant materials were obtained from plants cultivated specifically for experimental purposes, and no wild populations were disturbed. This complies with national biodiversity conservation and sustainability guidelines, especially considering the plant’s prior status as a Class II endangered species in Korea. To optimize extraction efficiency, preliminary experiments were conducted, and the final conditions involved reflux extraction with 70% ethanol (EtOH). In brief, 298.7 g of dried root powder was subjected to extraction with 3.5 L of 70% EtOH at 50 °C for 4 h. The resulting extract was filtered and concentrated under reduced pressure at 40 °C using a rotary evaporator (N-1200A, Eyela, Tokyo, Japan), yielding 93.3 g of crude extract, corresponding to a yield of 31.2% (*w*/*w*). To enrich for bioactive constituents and reduce non-specific cytotoxicity observed in the crude extract, solvent fractionation was performed based on polarity gradients. The ethanol extract was diluted in 1 L of distilled water and successively partitioned with n-hexane, ethyl acetate (EtOAc), and water-saturated n-butanol (W.S.-BuOH), resulting in four fractions. Specifically, this process yielded 0.30 g of n-hexane fraction (0.33%), 10.81 g of EtOAc fraction (11.59%), 33.60 g of W.S.-BuOH fraction (36.02%), and 43.46 g of aqueous fraction (46.59%). All fractions were dried under vacuum at 40 °C. Among these, the EtOAc fraction was selected for further investigation based on its superior bioactivity and relatively low cytotoxicity in preliminary screening assays.

#### Chemical Characterization of the EtOAc Fraction

The chemical composition of the ethyl acetate (EtOAc) fraction used in this study was previously analyzed and confirmed using liquid chromatography–mass spectrometry (LC-QTOF-MS and LC-MS/MS) and high-performance liquid chromatography (HPLC) methods, as reported in our prior publication [33].

The primary marker compounds, gallic acid and bergenin, were identified based on their retention times and mass fragmentation patterns.

Quantification revealed that gallic acid and bergenin were present at concentrations of 29.75 ± 0.10 mg/g extract and 123.12 ± 0.52 mg/g extract, respectively.

A summary of these results is provided in Supplementary Figure S1 and Table S1.

### 4.2. Reagents and Instruments

1-(4,5-Dimethylthiazol-2-yl)-3,5-diphenyl-formazan (MTT), fine dust (PM-10), and α-melanocyte-stimulating hormone (α-MSH) were purchased from Sigma-Aldrich (St. Louis, MO, USA). All other chemicals used in this study were of analytical grade and were obtained from Deajung (Siheung, Republic of Korea) and Junsei (Tokyo, Japan). Cell culture reagents, including Dulbecco’s Modified Eagle’s Medium (DMEM), Rosewell Park Memorial Institute medium, fetal bovine serum (FBS), and penicillin, were acquired from Biowest (Maine-et-Loire, France).

### 4.3. TYR Inhibition Activity

TYR inhibitory activity for skin whitening was measured according to the method described by Bernard et al. [45], with slight modifications. The EtOAc fraction of *A. tabularis* root and kojic acid as the positive control were diluted to various concentrations in a 96-well plate, followed by the addition of TYR (125 U). A substrate solution containing 10 mM L-3,4-dihydroxyphenylalanine (L-DOPA) dissolved in 67 mM phosphate-buffered saline (PBS) was added, and the reaction was carried out at 37 °C for 20 min. Absorbance was then measured at 490 nm using an enzyme-linked immunosorbent assay (ELISA) reader (Model 680, Bio-Rad, Hercules, CA, USA).

### 4.4. Elastase Inhibition Activity

Elastase inhibitory activity for preventing wrinkles was assessed based on the method described by Cannell et al. [46], with slight modifications. The EtOAc fraction of *A. tabularis* root and ursolic acid (positive control) were diluted in a 96-well plate, followed by the addition of elastase (0.25 U) dissolved in 100 mM tris-HCl buffer (pH 8.6). Subsequently, the substrate 1 mM N-succinyl-(l-ala)3-p-nitroanilide dissolved in 100 mM tris-HCl buffer (pH 8.6) was added, and the reaction was carried out at 37 °C for 20 min. Absorbance was then measured at 415 nm using an ELISA reader (Model 680, Bio-Rad, Hercules, CA, USA).

### 4.5. Cell Culture

All cell lines used in this study were obtained from the Korean Cell Line Bank (Seoul, Republic of Korea). Human immortalized keratinocytes (HaCaT) and murine metastatic melanoma cells (B16F10) were cultured in DMEM supplemented with 10% FBS and 1% penicillin (100 U/mL). Human skin fibroblasts (Detroit 551) were cultured in (minimal essential medium) MEM supplemented with 10% FBS and 1% penicillin (100 U/mL). All cells were cultured in a 5% CO_2_ incubator.

### 4.6. MTT Assay

The cell viability of the *A. tabularis* root EtOAc fraction was assessed using the MTT assay described by Mosmann et al. [47], with minor modifications. Briefly, the cells were seeded into 96-well plates at a density of 1 × 10^4^ cells/well and incubated at 37 °C and 5% CO_2_ for 24 h. After incubation, the media were removed, and the cells were treated with various concentrations of the sample (10, 25, 50, 75, 100, and 200 µg/mL). Following a 24 h incubation, the medium was removed, and the cells were treated with MTT solution diluted to 500 µg/mL in PBS. After 4 h, the MTT solution was removed, DMSO was added, and absorbance was measured at 540 nm using an ELISA reader (Model 680, Bio-Rad, Hercules, CA, USA).

### 4.7. Measurement of Melanin Content

B16F10 cells were seeded into 6-well plates at 2 × 10^5^ cells/well. After 24 h, the cells were treated with 1 mM α-MSH and various concentrations of the EtOAc fraction of *A. tabularis* root. After 72 h of incubation, melanin production was evaluated. The cells were harvested and centrifuged, after which the cell pellet was treated with 200 µL of 1 N NaOH, heated at 100 °C for 10 min, and then cooled on ice. Subsequently, absorbance was measured at 475 nm using an ELISA reader (Model 680, Bio-Rad, Hercules, CA, USA).

### 4.8. Fine Dust Preparation

Fine dust (PM-10) was dispersed in sterilized distilled water at a concentration of 10,000 μg/mL and sonicated for 40 min to prevent aggregation. Subsequently, the fine dust was diluted to a final concentration of 75 µg/mL in serum-free DMEM or MEM before use.

### 4.9. Culture and Treatment of 3D Artificial Skin

Three-dimensional (3D) artificial skin models, Neoderm-ED (epidermis + dermis) and Neoderm-ME (epidermis + melanocytes), were procured from Tego-Science (Seoul, Republic of Korea). The artificial skin samples were treated with fine dust or α-MSH for 24–72 h according to the experimental conditions and incubated at 37 °C in an atmosphere containing 5% CO_2_. The samples were then fixed in 4% formalin for hematoxylin and eosin (H&E) staining and immunohistochemical analysis.

### 4.10. Evaluation of Primary Skin Irritation Using Neoderm-ED and Neoderm-ME

Neoderm-ED and Neoderm-ME were washed twice with PBS, transferred into 12-well plates containing Neoderm media, and preincubated for 24 h at 37 °C in an atmosphere containing 5% CO_2_. The samples were then treated with various concentrations of *A. tabularis* root EtOAc fraction (10, 25, 50 and 75 µg/mL) and incubated for another 24 h. Following incubation, the samples were treated with MTT solution (500 µg/mL) for 4 h. Subsequently, the media were removed, DMSO was added, and absorbance was measured at 540 nm using an ELISA reader (Model 680, Bio-Rad, Hercules, CA, USA). Cell viability below 50% was considered indicative of irritation [48,49]. The untreated skin equivalents cultured under identical conditions served as the negative control group.

### 4.11. Real-Time PCR

Real-time polymerase chain reaction (RT-PCR) analysis was performed to examine the effects of *A. tabularis* root EtOAc fraction on mRNA expression levels of skin-related factors induced by fine dust and melanin-related factors. The cells were seeded into 6-well plates at a density of 2.5 × 10^5^ cells/well and incubated at 5% CO_2_ for 24 h. Subsequently, artificial skin models were preincubated. To evaluate the expression of skin-related factors induced by fine dust, the media were replaced with samples at various concentrations mixed with 75 ppm of fine dust and incubated for 24 h at 5% CO_2_. Similarly, to examine melanin-related factors, the media were replaced with various concentrations of samples mixed with 1 mM α-MSH and incubated for 72 h at 5% CO_2_. mRNA was extracted using an RNA-Spin™ Total RNA Extraction Kit (iNtRON Biotechnology, Anyang, Republic of Korea), after which cDNA was synthesized using PrimeScript™ RT Master Mix (Takara Bio, Shiga, Japan). RT-PCR was performed using SYBR Green (Enzynomics, Daejeon, Republic of Korea), 1 µL each of forward and reverse primers, and RNase-free distilled water, mixed with 1 µL cDNA. Real-time PCR was performed on a CronoSTAR™ 96 Real-Time PCR System (Clontech, Palo Alto, CA, USA) under the following conditions: 95 °C for 10 s, 60 °C for 15 s, 72 °C for 15 s, repeated for 45 cycles. GAPDH was used as the internal control. The primer sequences for whitening-related experiments are shown in Table 1, and those for the wrinkle improvement and skin barrier experiments are listed in Table 2.

### 4.12. Statistical Analysis

All statistical comparisons were conducted using a two-tailed unpaired Student’s *t*-test, as each experiment involved a direct comparison between the control and a single treatment group. All measurements were performed in triplicate, and the results are expressed as the mean ± standard deviation (SD). Statistical analysis was performed using IBM SPSS Statistics version 24 (IBM Corp., Armonk, NY, USA). A *p*-value < 0.05 was considered statistically significant. In addition, correlation analyses between gene expression levels were conducted using MetaboAnalyst version 6.0.

## 5. Conclusions

The ethyl acetate (EtOAc) fraction derived from *Astilboides tabularis* root demonstrated multifunctional cosmetic properties, including skin-whitening, antiwrinkle, and moisturizing effects. The fraction significantly inhibited melanogenesis by suppressing tyrosinase activity and the expression of key melanogenesis-related genes (MITF, TYR, TRP-1, and TRP-2). It also improved skin hydration and barrier function by upregulating the expression of filaggrin, AQP-3, HAS-1, and HAS-2 while promoting collagen (COL1A1) and elastin synthesis. Simultaneously, it inhibited wrinkle formation by reducing the expression of matrix metalloproteinases (MMP-1, MMP-3, MMP-9) and enhancing TIMP-1 and TIMP-2 expression. These effects were validated in both cell-based assays and advanced 3D artificial skin models (Neoderm-ED and Neoderm-ME). Furthermore, primary skin irritation testing confirmed its safety for topical application. The observed bioactivities are likely attributable to the combined actions of bergenin and gallic acid, the primary active compounds identified in the fraction. Overall, this study highlights the EtOAc fraction of *A. tabularis* root as a promising, safe, and effective natural ingredient for skincare applications. Further clinical studies are recommended to substantiate its efficacy in human subjects.

## Figures and Tables

**Table 1:** F10 cells and Neoderm-ME used in the RT-PCR analysis.

Primer Name	Sequences	
GADPH	Forward	5′-GGTTGTCTCCTGCGACTTCA-3′
	Reverse	5′-TGGTCCAGGGTTTCTTACTCC-3′
MITF	Forward	5′-CATCATCAGCCTGGAATCAA-3′
	Reverse	5′-TCAAGTTTCCAGAGACGGGT-3′
TYR	Forward	5′-TCTTCACCATGCTTTTGTGG-3′
	Reverse	5′-ATAGGTGCATTGGCTTCTGG-3′
TRP-1	Forward	5′-TGGTCTGTGAATCCTTGGAA-3′
	Reverse	5′-CATTTCCAGCTGGGTTTCTC-3′
TRP-2	Forward	5′-CGTGCTGAACAAGGAATGC-3′
	Reverse	5′-CGAAGGATATAAGGGCCACTC-3′

**Table 2:** HaCaT cells and Neoderm-ED used in the RT-PCR analysis.

Primer Name	Sequences	
GADPH	Forward	5′-CAATGAATACGGCTACAGCAAC-3′
	Reverse	5′-AGGGAGATGCTCAGTGTTGG-3′
MMP-1	Forward	5′-ACCAAGGAGCGAAGATAG-3′
	Reverse	5′-CAGGCGGAGTATGAGATAA-3′
MMP-3	Forward	5′-ACAAAGGATACAACAGGGACCAA-3′
	Reverse	5′-CCAGGGAGTGGCCAATTTC-3′
MMP-9	Forward	5′-CCCACTGCTGGCCCTTCTA-3′
	Reverse	5′-TCACGTTGCAGGCATCGT-3′
COL1A1	Forward	5′-AGGGCCAAGACGAAGACATC-3′
	Reverse	5′-AGATCACGTCATCGCACAACA-3′
HAS1	Forward	5′-GTGCGGGTACTGGACGA-3′
	Reverse	5′-GACCGCTGATGCAGGATACA-3′
HAS2	Forward	5′-GCAGTGTAAGATATTGGATGGC-3′
	Reverse	5′-CCCATAAATTCTTGATTGTACCAATCTTC-3′
AQP3	Forward	5′-GGGACCAGTCGGAAGGGAT-3′
	Reverse	5′-CACAGATGGACAGGCTGCCT-3′
Elastin	Forward	5′-CTTCAGAGCAGTTCCCATTC-3′
	Reverse	5′-AATCCCCAAATATCCAGGACAA-3′
TIMP-1	Forward	5′-TGACATCCGGTTCGTCTACA-3′
	Reverse	5′-TGCAGTTTTCCAGCAATGAG-3′
TIMP-2	Forward	5′-GCGGTCAGTGAGAAGGAAGTGGA-3′
	Reverse	5′-GAGGAGGGGGCCGTGTAGATAAAC-3′
Filaggrin	Forward	5′-AAGCTTCATGGTGATGCGAC-3′
	Reverse	5′-TCAAGCAGAAGAGGAAGGCA-3′

## Data Availability

All data generated or analyzed during this study are included in this published article.

## Supplementary Materials

The following supporting information can be downloaded at https://www.mdpi.com/article/10.3390/ijms26125725/s1.

## Abbreviations

The following abbreviations are used in this manuscript:

3D	three-dimensional
AQP-3	aquaporin-3
COL1A1	collagen type I alpha 1 chain
EtOAc	ethyl acetate
H&E	hematoxylin and eosin
HAS	hyaluronan acid synthase
MITF	microphthalmia-associated transcription factor
MMP	matrix metalloproteinase
PM-10	particulate matter ≤10 microns
RT-PCR	real-time polymerase chain reaction
TIMP	tissue inhibitor of metalloproteinases
TRP	tyrosinase-related protein
TYR	tyrosinase
α-MSH	alpha-melanocyte stimulating hormone
