# Supplementary material for: Skin-Whitening, Antiwrinkle, and Moisturizing Effects of Astilboides tabularis (Hemsl.) Engl. Root Extracts in Cell-Based Assays and Three-Dimensional Artificial Skin Models
Source: Int J Mol Sci. 2025 Jun 15;26(12):5725. doi: 10.3390/ijms26125725 (PMC12193470; doi:10.3390/ijms26125725)
Supplement: Supplementary file 1 [file ijms-26-05725-s001.zip › ijms-3674004-supplementary.pptx]

## Slide 1
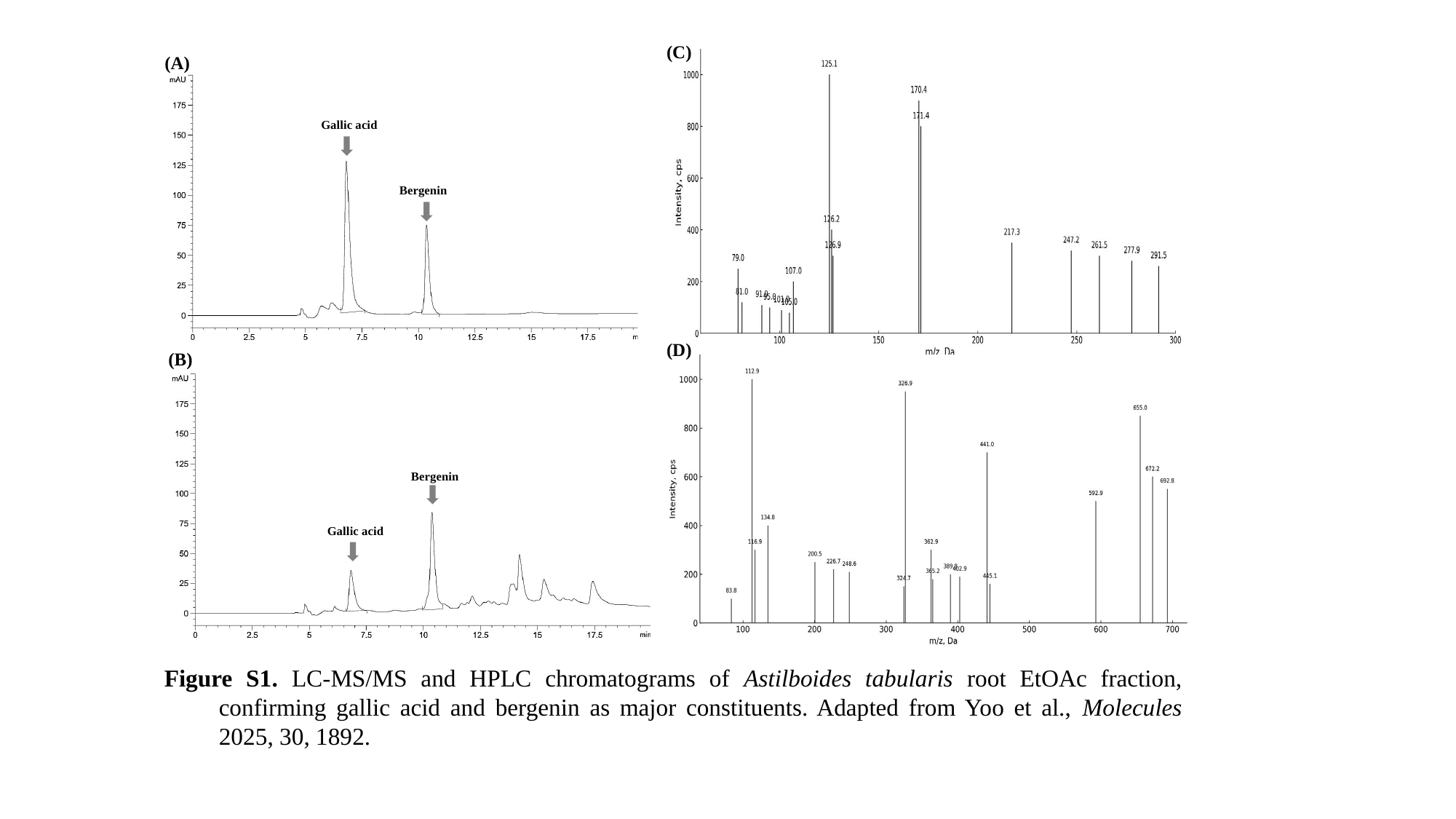

(C)
(A)
Gallic acid
Bergenin
(D)
(B)
Bergenin
Gallic acid
Figure S1. LC-MS/MS and HPLC chromatograms of Astilboides tabularis root EtOAc fraction, confirming gallic acid and bergenin as major constituents. Adapted from Yoo et al., Molecules 2025, 30, 1892.

## Slide 2
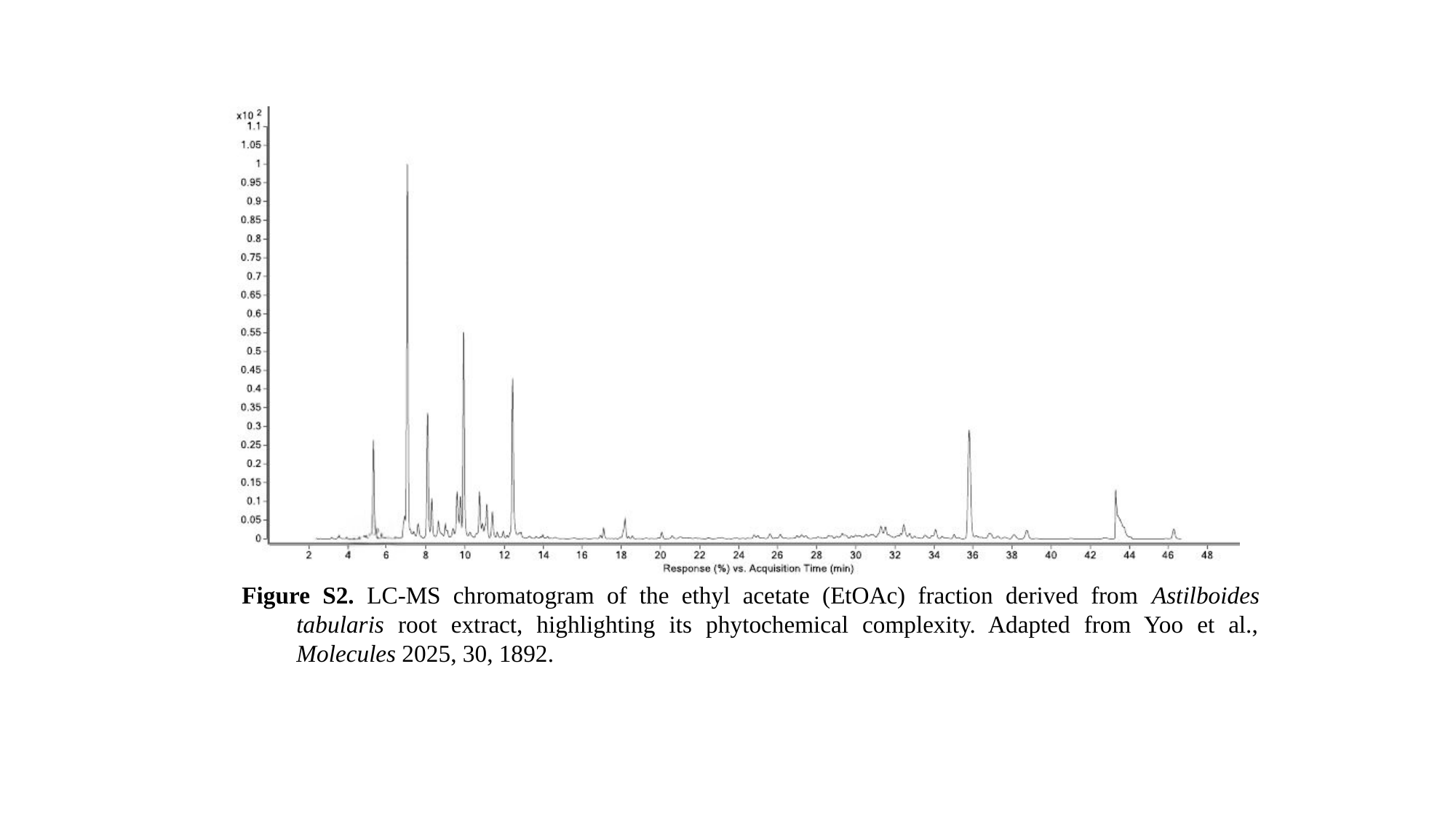

Figure S2. LC-MS chromatogram of the ethyl acetate (EtOAc) fraction derived from Astilboides tabularis root extract, highlighting its phytochemical complexity. Adapted from Yoo et al., Molecules 2025, 30, 1892.

## Slide 3
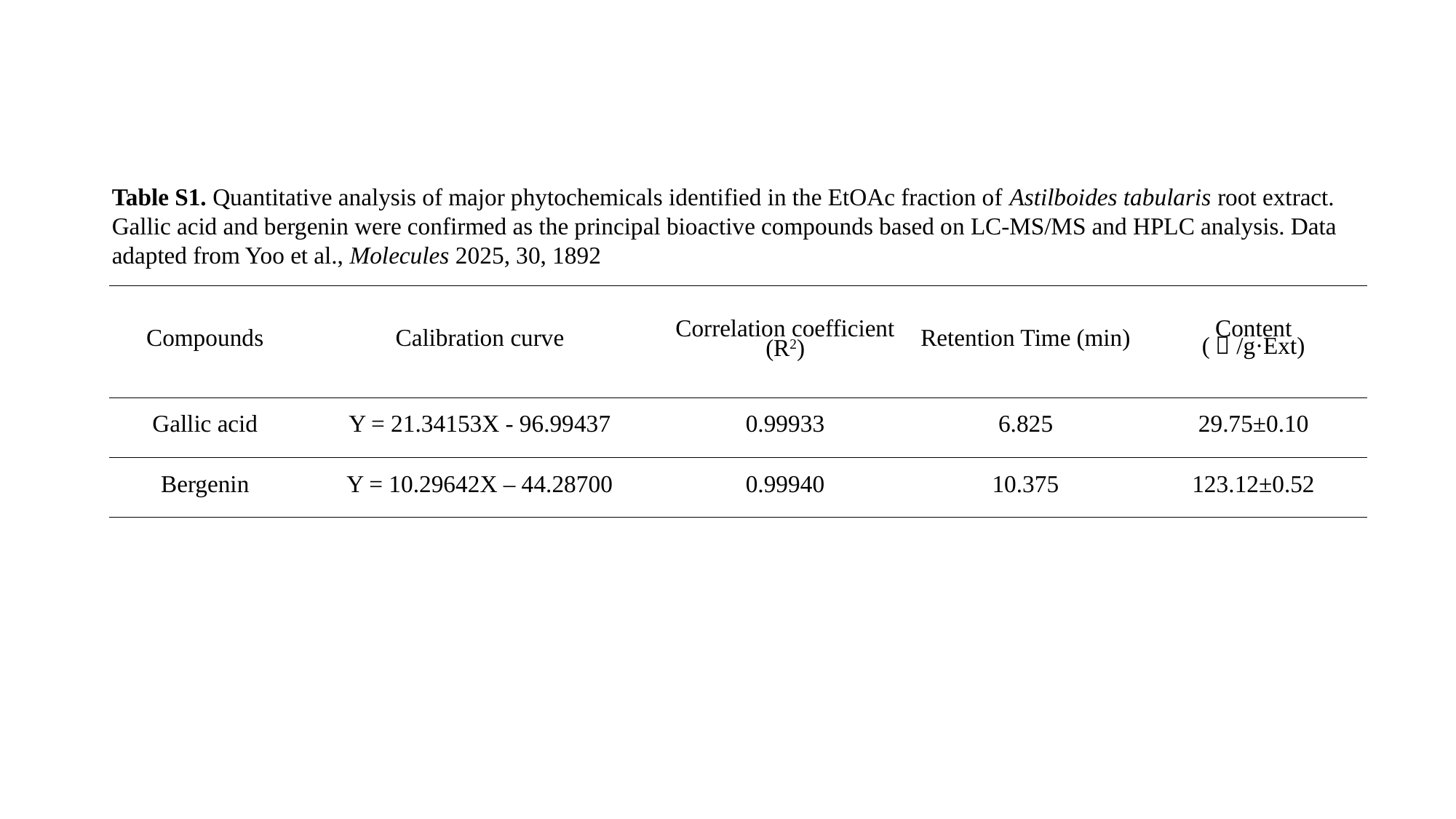

Table S1. Quantitative analysis of major phytochemicals identified in the EtOAc fraction of Astilboides tabularis root extract. Gallic acid and bergenin were confirmed as the principal bioactive compounds based on LC-MS/MS and HPLC analysis. Data adapted from Yoo et al., Molecules 2025, 30, 1892
| Compounds | Calibration curve | Correlation coefficient (R2) | Retention Time (min) | Content (㎎/g·Ext) |
| --- | --- | --- | --- | --- |
| Gallic acid | Y = 21.34153X - 96.99437 | 0.99933 | 6.825 | 29.75±0.10 |
| Bergenin | Y = 10.29642X – 44.28700 | 0.99940 | 10.375 | 123.12±0.52 |
